# Anti-Inflammatory Effect of 4,5-Dicaffeoylquinic Acid on RAW264.7 Cells and a Rat Model of Inflammation

**DOI:** 10.3390/nu13103537

**Published:** 2021-10-09

**Authors:** Goeun Jang, Seulah Lee, Joonho Hong, Boram Park, Dokyung Kim, Chunsung Kim

**Affiliations:** 1 Department of Oral Biochemistry, College of Dentistry, Chosun University, Gwangju 61452, Korea; goeun2748@gmail.com (G.J.); seulah21@naver.com (S.L.); 2 Nano Bio Research Center, Jeonnam Bioindustry Foundation, Jangseong-si 57248, Jeollanam-do, Korea; jhhong7912@jbf.kr; 3 Department of Dental Hygiene, College of Health and Welfare, Kyungwoon University, Gumi-si 39160, Gyeongsangbuk-do, Korea; br8288@naver.com; 4 Oral Biology Research Institute, College of Dentistry, Chosun University, Gwangju 61452, Korea; kdk@chosun.ac.kr

**Keywords:** 4,5-dicaffeoylquinic acid, anti-inflammation, carrageenan

## Abstract

Anti-inflammatory agents that are safer and more effective than the currently used non-steroidal anti-inflammatory drugs are urgently needed. The dicaffeoylquinic acid (diCQA) isomer 4,5-diCQA exhibits antioxidant activity and various other health-promoting benefits; however, its anti-inflammatory properties require further investigation. This study was conducted to evaluate the anti-inflammatory properties of 4,5-diCQA in vitro and in vivo using RAW264.7 cells and a carrageenan-induced inflammation model, respectively. In RAW264.7 cells, 4,5-diCQA pretreatment significantly inhibited lipopolysaccharide-induced expression of nitric oxide, prostaglandin E_2_, nitric oxide synthase, cyclooxygenase-2, tumor necrosis factor-α, interleukin-1β, and interleukin-6, without inducing cytotoxicity. The inhibitory effects of 4,5-diCQA were mediated by the suppression of nuclear factor-κB nuclear translocation and mitogen-activated protein kinase (MAPK) phosphorylation. Oral administration of 4,5-diCQA at doses of 5, 10, and 20 mg/kg of the body weight suppressed carrageenan-induced edema and the expression of nitric oxide synthase, cyclooxygenase-2, and tumor necrosis factor-α in a dose-dependent manner. Collectively, our results suggest that 4,5-diCQA exerts anti-inflammatory effects by suppressing activation of the nuclear factor-κB and MAPK pathways in vitro and reducing carrageenan-induced edema in vivo. Therefore, 4,5-diCQA shows potential as a natural alternative to non-steroidal anti-inflammatory drugs.

## 1. Introduction

Inflammation is a complex, essential defense system that responds to biological, chemical, and physical stimuli. Inflammation is initiated and regulated by interactions between cellular and molecular factors [1,2,3]. In the acute phase of the inflammatory response, immune cells migrate to damaged tissues and promote healing by releasing soluble mediators such as cytokines, chemokines, and acute phase proteins [1,2,3,4,5]. Persistent inflammation due to long-term irritation or inappropriate cellular reactions causes tissue damage and fibrosis, leading to chronic inflammatory diseases such as arthritis, asthma, atherosclerosis, diabetes, and cancer [1,2,6,7,8]. Therefore, it is important to properly regulate inflammatory responses. Currently, non-steroidal anti-inflammatory drugs are widely used to treat inflammation; however, cyclooxygenase (COX)-1 causes gastrointestinal disorders and the use of COX-2 is limited in cases of cardiovascular disorders [9,10]. Therefore, safer and more effective anti-inflammatory agents are urgently needed.

The abundant phytochemical components in plants, such as polyphenols and flavonoids, can relieve inflammation and treat related diseases, based on which various drugs have been developed [11,12]. Among these, chlorogenic acid and mono- and dicaffeoyl esters of quinic acid have attracted attention because of their antioxidant, antiviral, antibacterial, and anticancer activities [12,13,14,15]. The structure of dicaffeoylquinic acid (diCQA) contains two caffeic acid molecules linked to one quinic acid molecule via an ester bond. Numerous isomers of 3,5-diCQA, 3,4-diCQA, and 4,5-diCQA have been reported [15]. The latter has superior antioxidant activity compared to the other isomers and chlorogen, which are well-known antioxidants [12,16,17]. In addition to its high level of antioxidant activity, 4,5-diCQA exhibits anti–prostate cancer, anti-diabetes, and cognition-enhancing properties [12,18]. However, other physiological activities have not been clarified. Therefore, in the present study, we aimed to verify the anti-inflammatory effects of 4,5-diCQA and underlying mechanisms in vivo and in vitro.

## 2. Materials and Methods

### 2.1. Reagents

Lipopolysaccharide (LPS), carrageenan, 4,5-diCQA, 3-(4,5-dimethylthiazol-2-yl)-2,5-diphenyltetrazolium bromide (MTT), phosphoric acid, sulfanilamide, and *N*-(1-naphthyl)ethylenediamine dihydrochloride were purchased from Sigma–Aldrich (St. Louis, MO, USA). A prostaglandin E_2_ enzyme-linked immunosorbent assay (PGE_2_ ELISA) kit was purchased from R&D Systems (Minneapolis, MN, USA). All antibodies were purchased from Cell Signaling Technology (Danvers, MA, USA), except for anti-inducible NO synthase (iNOS) (Abcam, Cambridge, UK), anti-NF-κB(p65), and anti-α-tubulin (Thermo Fisher Scientific, Waltham, MA, USA). Dulbecco’s modified Eagle’s medium and penicillin/streptomycin solution were purchased from WelGene (Daegu, Korea). Fetal bovine serum was purchased from ATLAS Biologicals (Fort Collins, CO, USA).

### 2.2. Cell Culture

Murine macrophage RAW264.7 cells were obtained from the Korea Research Institute of Bioscience and Biotechnology (Daejeon, Korea). Cells were cultured in Dulbecco’s modified Eagle’s medium containing 10% fetal bovine serum and 1% penicillin/streptomycin in a humidified incubator with 5% CO_2_ at 37 °C.

### 2.3. Cell Viability

The cytotoxicity of 4,5-diCQA to RAW264.7 cells was determined via the MTT assay according to the manufacturer’s protocol. Briefly, the cells were seeded at 1 × 10^6^ cells/mL in 12-well plates and were incubated with different concentrations of 4,5-diCQA (0, 2.5, 5, 10, 20, and 40 µM) for 24 h. Following incubation, 100 µL of MTT solution (5 mg/mL) was added to each well, and the cells were incubated for an additional 4 h at 37 °C. After removing the culture medium containing the MTT solution, 1 mL of dimethyl sulfoxide was added to each well, and the absorbance was measured at 590 nm using a microplate reader (Epoch Biotek Instruments, Inc., Winooski, VT, USA). The experiments were performed in triplicate. The cell viability was calculated with the average absorbance of the negative control group as 100%, and the formula is as follows:

$$\mathrm{Cell}\;\mathrm{viability}\;\left(\%\right)=\frac{\mathrm{Absorbance}\;\mathrm{of}\;\mathrm{Treat}\;\mathrm{group}}{\mathrm{Average}\;\mathrm{absorbance}\;\mathrm{of}\;\mathrm{control}\;\mathrm{group}}\times 100$$

### 2.4. Measurement of Nitrite and PGE_2_

RAW264.7 cells (1 × 10^6^ cells/mL in 6-well plates) were pretreated with 4,5-diCQA for 1 h and stimulated with LPS (50 ng/mL) for 24 h. Nitrite accumulation in the culture medium was measured using Griess reagent. Briefly, 100 µL of culture medium was mixed with 100 µL of Griess reagent (1% (*v*/*v*) sulfanilamide and 0.1% (*w*/*v*) naphthylethylenediamine in 5% (*v*/*v*) phosphoric acid). The absorbance was measured at 540 nm using a microplate reader (Epoch Biotek Instruments, Inc.). Nitrite was quantified using a standard sodium nitrite curve. PGE_2_ accumulation in the culture medium was quantified using a PGE_2_ Parameter™ PGE_2_ assay kit (R&D Systems) according to the manufacturer’s instructions.

### 2.5. Western Blot Analysis

RAW264.7 cells were pretreated with 4,5-diCQA for 1 h and stimulated with LPS (50 ng/mL) for 1 or 24 h. The cells were washed twice with ice-cold PBS and lysed using PRO-PREP protein extraction solution (iNtRON Biotechnology, Seongnam, Gyeonggi, Korea) to extract total protein. Cytoplasmic and nuclear proteins were prepared using NE-PER™ nuclear and cytoplasmic extraction reagents (Thermo Fisher Scientific, Waltham, MA, USA) according to the manufacturer’s protocol. Spinal cord tissue was homogenized using PRO-PREP buffer to extract the proteins. Protein concentrations were quantified using a bicinchoninic acid protein assay kit (Pierce, Rockford, IL, USA). Equivalent amounts (20 μg) were separated on 8%, 10%, or 15% sodium dodecyl sulfate-polyacrylamide gels and transferred to polyvinylidene difluoride membranes (Bio-Rad Laboratories, Hercules, CA, USA). The transblotted membranes were blocked with 5% bovine serum albumin in Tris-buffered saline containing 0.1% Tween 20 for 30 min, followed by overnight incubation with primary antibodies at 4 °C. The membranes were incubated with horseradish peroxidase–linked secondary antibody (1:2500) for 1 h. Protein bands were detected using an enhanced chemiluminescence kit (Millipore, Billerica, MA, USA) and visualized using a MicroChemi 4.2 imager (DNR Bioimaging Systems, Jerusalem, Israel).

### 2.6. Animals

Male specific pathogen-free Sprague–Dawley rats, weighing 200–220 g, were purchased from Damool Science (Daejeon, Korea). The animals were housed in a controlled environment (temperature: 21 ± 1 °C; humidity: 55 ± 5%; 12-h light/dark cycle) and were allowed free access to commercial pellets and water. All animal-handling procedures were performed in accordance with the National Institutes of Health Guide for the Care and Use of Laboratory Animals [19]. The experimental protocol was approved by the Chosun University Institutional Animal Care and Use Committee (CIACUC2021-S0016).

### 2.7. Experimental Design and Drug Treatment

The inhibitory effect of 4,5-diCQA on carrageenan-induced rat paw edema was evaluated with minor modifications as described by Akinnawo et al. [20]. The rats (n = 24) were randomly divided into six groups: group 1 (non-inflamed, 0.9% normal saline, as the negative control), group 2 (paw edema, 0.9% normal saline), group 3–5 (paw edema, 5, 10, and 20 mg/kg 4,5-diCQA, respectively), and group 6 (paw edema, 10 mg/kg diclofenac sodium, as the positive control). Edema was induced by the injection of 1% (*w*/*v*) carrageenan suspension (0.1 mL) in 0.9% normal saline into the sub-plantar surface of both hind limbs. The treated groups were orally administered 4,5-diCQA and diclofenac sodium 1 h prior to injecting carrageenan. The thickness of the middle of the paw was measured using a digital caliper at specific time intervals, from 0 (before carrageenan injection) to 24 h after injection. The inhibition rate of paw edema was calculated using the following formula:

$$\mathrm{Inhibition}\;\mathrm{rate}\left(\%\right)=\frac{{\left(C_{t}-C_{0}\right)}_{\mathrm{control}}-{\left(C_{t}-C_{0}\right)}_{\mathrm{treated}}}{{\left(C_{t}-C_{0}\right)}_{\mathrm{control}}}\times 100$$

where C_t_ = paw thickness (mm) of the left hind limb at time t, C_0_ = paw thickness (mm) of the left hind limb before carrageenan injection, (C_t_ − C_0_)_control_ = increase in rat paw size after injecting carrageenan at time t, and (C_t_ − C_0_)_treated_ = increase in paw size after injecting carrageenan into control or treated rats at time t.

### 2.8. Statistical Analyses

Data are shown as the mean ± standard deviation (SD). One-way analysis of variance followed by Tukey’s test was used for multigroup comparisons using GraphPad Prism 5.0 software (GraphPad, Inc., San Diego, CA, USA). Statistical significance was set at *p* < 0.05.

## 3. Results

### 3.1. Effect of 4,5-diCQA on RAW264.7 Cell Viability

**Figure 1:**
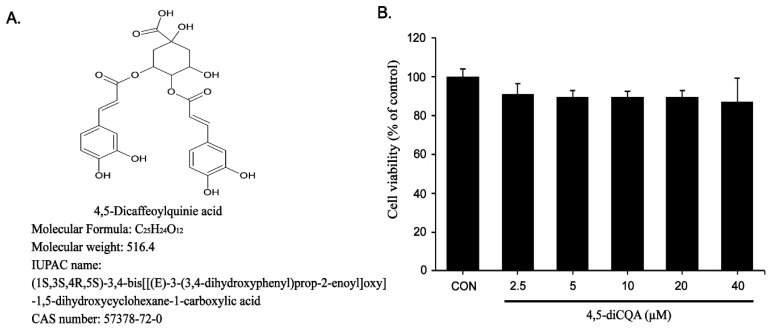
Effects of 4,5-diCQA on RAW264.7 cells viability. (**A**) Chemical formula of 4,5-diCQA. (**B**) Cells were treated with 4,5-diCQA (2.5, 5, 10, 20, and 40 µM) for 24 h, and viability was determined by MTT assay. Cells incubated without 4,5-diCQA were used as controls and considered as 100% viable. Data are represented as the mean ± SD of three independent experiments. CON; control.

Prior to assessing the anti-inflammatory effects of 4,5-diCQA, cytotoxicity was evaluated using the MTT assay. RAW264.7 cells were treated with various concentrations of 4,5-diCQA (0, 2.5, 5, 10, 20, and 40 µM) for 24 h. No cytotoxicity was observed at concentrations up to 40 µM (Figure 1B).

### 3.2. Inhibitory Effects of 4,5-diCQA on Nitrite Production and Anti-Inducible NO Synthase (iNOS) Protein Expression in RAW264.7 Cells Stimulated with Lipopolysaccharide (LPS)

**Figure 2:**
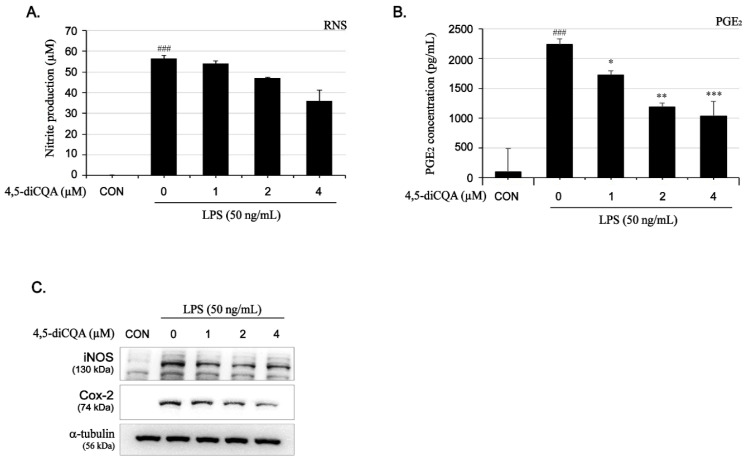
Inhibitory effects of 4,5-diCQA on LPS-induced nitrite, PGE_2_, iNOS, and COX-2 in RAW264.7 cells-stimulated with LPS. Cells were pretreated with 4,5-diCQA (1, 2, and 4 µM) for 1 h, followed by LPS (50 ng/mL) stimulation for 24 h. (**A**) Nitrite production was determined in the culture medium using Griess reagent. (**B**) PGE_2_ production was determined in the culture medium using enzyme-linked immunosorbent assay (ELISA). (**C**) Expression of iNOS and COX-2 was determined using Western blot analysis. α-Tubulin served as an internal control. Expression results are represented as the mean ± SD of three independent experiments. ### *p* < 0.01 compared with control group; * *p* < 0.05, ** *p* < 0.01, and *** *p* < 0.001 compared with LPS-treated group. CON; control, RNS; reactive nitrogen species, PGE_2_; prostaglandin E_2_, iNOS; inducible nitrite oxide, COX-2; cyclooxygenase-2.

To verify the anti-inflammatory effects of 4,5-diCQA, nitrite production and iNOS expression were evaluated using Griess reagent and Western blotting, respectively. As shown in Figure 2A,C, LPS significantly induced nitrite production (56-fold, 56.6 ± 1.5 µM) compared to that in the control (1.3 ± 0.3 µM). However, pretreatment with 4,5-diCQA reduced nitrite production in a dose-dependent manner (Figure 2A). Consistent with the nitrite production results, LPS significantly increased iNOS protein expression, but this effect decreased gradually in response to 4,5-diCQA pretreatment in a concentration-dependent manner (Figure 2C).

### 3.3. Inhibitory Effects of 4,5-diCQA on PGE_2_ Production and COX-2 Protein Expression in RAW264.7 Cells Stimulated with LPS

As shown in Figure 2B, LPS markedly increased PGE_2_ production (22-fold, 2237.9 ± 90.71 µM) compared to in the control (99.9 ± 393 µM); however, pretreatment with 4,5-diCQA significantly suppressed LPS-induced PGE_2_ production in a dose-dependent manner, with an inhibition rate of 55% at 4 µM. Additionally, LPS significantly increased COX-2 protein expression, but this effect decreased gradually in response to 4,5-diCQA pretreatment in a concentration-dependent manner (Figure 2C). These results suggest that 4,5-diCQA potently inhibits PGE_2_ production and COX-2 protein expression.

### 3.4. Inhibitory Effects of 4,5-diCQA on Pro-Inflammatory Cytokines in RAW264.7 Cells Stimulated with LPS

**Figure 3:**
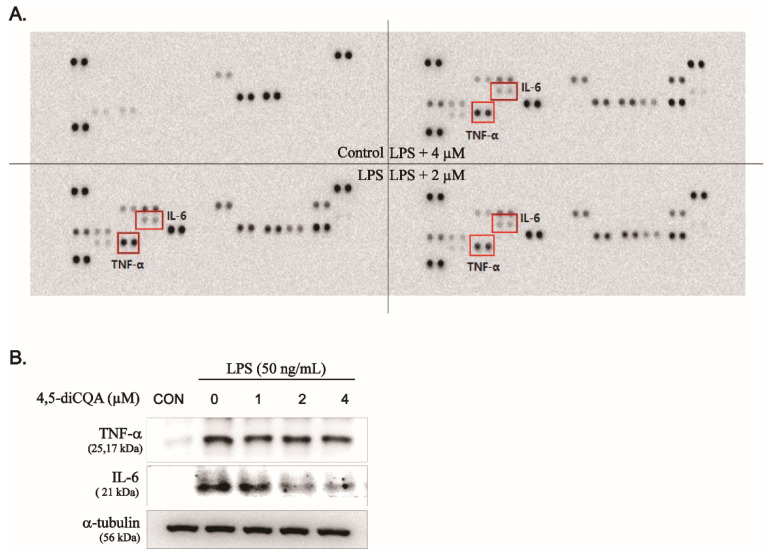
Inhibitory effect of 4,5-diCQA on LPS-induced TNF-α and IL-6 in RAW264.7 cells-stimulated with LPS. Cells were pretreated with 4,5-diCQA (1, 2, and 4 µM) for 1 h, followed by LPS (50 ng/mL) stimulation for 24 h. (**A**) TNF-α and IL-6 were determined in the cultured medium using mouse cytokines array. (**B**) Expression of TNF-α and IL-6 was determined using Western blot analysis. α-Tubulin served as an internal control. CON; control, TNF-α; tumor necrosis factor-alpha, IL-6; interleukin-6.

We investigated the effect of 4,5-diCQA on LPS-induced expression of pro-inflammatory cytokines using a mouse cytokine array and Western blotting. As shown in Figure 3A, LPS significantly induced the expression of various immune- and inflammation-related factors. Among them, the expression of IL-6 and TNF-α, which play important roles in the inflammatory response, were significantly decreased by 4,5-diCQA; the inhibition rates were 20% and 40% at 4 µM, respectively (Figure 3A). Furthermore, Western blotting showed that LPS-induced protein expression of IL-6 and TNF-α was decreased by 4,5-diCQA in a dose-dependent manner (Figure 3B). These results suggest that 4,5-diCQA suppressed LPS-induced IL-6 and TNF-α protein expression, supporting the hypothesis that 4,5-diCQA has anti-inflammatory activity.

### 3.5. Suppression of NF-κB and MAPK Signaling Pathways by 4,5-diCQA in RAW264.7 Cells Stimulated with LPS

**Figure 4:**
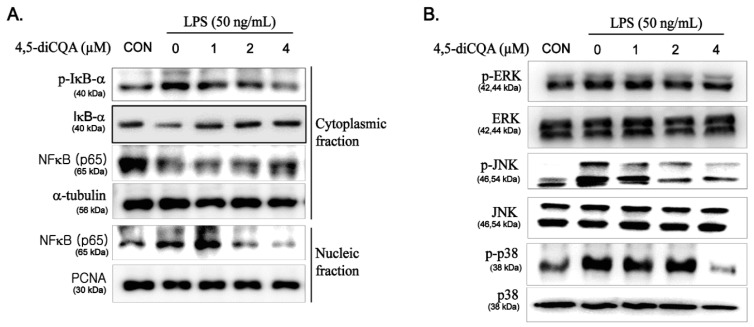
Effects of 4,5-diCQA on LPS-induced activation of NF-κB and phosphorylation of MAPKs in RAW264.7 cells-stimulated with LPS. Cells were pretreated with 4,5-diCQA (1, 2, and 4 µM) for 1 h, followed by LPS (50 ng/mL) stimulation for 1 h. (**A**) Phosphorylation levels of IκB-α and NF-κB p65 translocation to nucleus were determined using Western blot analysis. (**B**) Protein levels of phosphorylation of MAPKs (ERK, JNK, and p38) were determined using Western blot analysis. α-Tubulin and Lamin B1 were used as cytosolic and nuclear internal controls, respectively. CON; control, IκB-α; nuclear factor of kappa light polypeptide gene enhancer in B-cells inhibitor, alpha, p65; NF-kappa-B p65 subunit, ERK; extracellular signal-regulated kinase, JNK; c-Jun N-terminal kinase.

The critical role of the NF-κB and MAPK signaling pathways in regulating the expression of inflammatory mediators is well-known; thus, inhibition of these pathways is generally regarded as an important mechanism in the inflammatory response. We examined whether the anti-inflammatory effect of 4,5-diCQA on inflammatory mediators and cytokines was affected by kinases in these pathways. As shown in Figure 4A, LPS stimulation induced the phosphorylation and degradation of IκB-α in the cytoplasm, thereby enhancing translocation of NF-κB p65 from the cytoplasm to the nucleus. However, pretreatment with 4,5-diCQA inhibited LPS-induced nuclear translocation of NF-κB p65 by suppressing the phosphorylation and degradation of IκB-α. In addition, LPS alone induced phosphorylation of ERK, JNK, and p38 MAPK without affecting their total protein levels, whereas pretreatment with 4,5-diCQA significantly suppressed this effect in a dose-dependent manner (Figure 4B). These results suggest that the NF-κB and MAPK pathways mediate the anti-inflammatory effect of 4,5-diCQA in RAW264.7 cells stimulated by LPS.

### 3.6. Anti-Inflammatory Effects of 4,5-diCQA In Vivo

**Figure 5:**
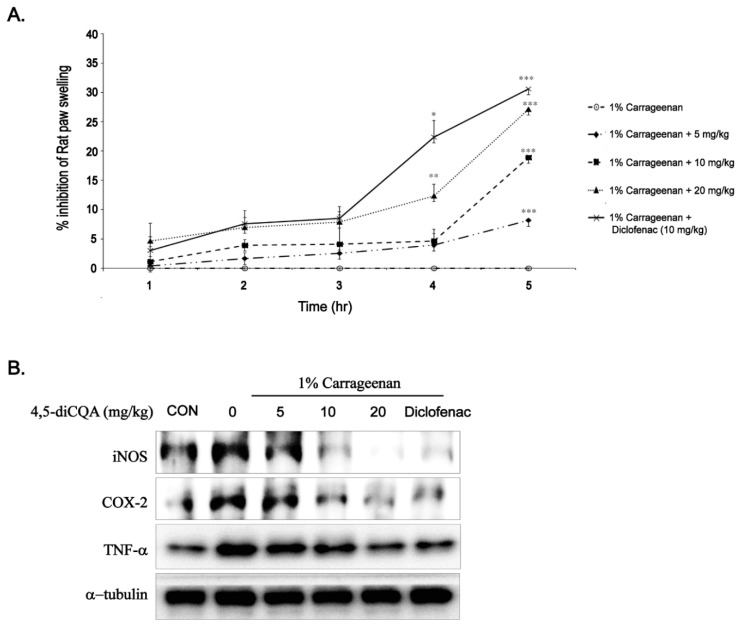
Effects of 4,5-diCQA on carrageenan-induced paw edema. Paw edema was induced by subcutaneous injection of a 1% carrageenan solution (100 µL/animal) into the hind paw, 1 h after oral administration of 4,5-diCQA (5, 10, and 20 mg/kg) or diclofenac sodium (10 mg/kg). (**A**) Paw thickness was measured at 0–5 h after carrageenan injection. Results are the means ± SD of four animals. * *p* < 0.05, ** *p* < 0.01, and *** *p* < 0.001 compared to the carrageenan group at the corresponding time points. (**B**) At the end point, expression of iNOS, COX-2, and TNF-α in the spinal cord of experimental animals was determined using Western blot analysis. α-Tubulin served as an internal control. CON; control, iNOS; inducible nitrite oxide, COX-2; cyclooxygenase-2, TNF-α; tumor necrosis factor-alpha.

To verify the anti-inflammatory effect of 4,5-diCQA in vivo, suppression of carrageenan-induced paw edema was assessed. As shown in Figure 5A, the inhibition rate of paw edema thickness by 4,5-diCQA (5, 10, and 20 mg/kg) and diclofenac sodium (positive control, 10 mg/kg) gradually increased over 5 h compared with that in the untreated control group. Furthermore, at 5 h, the effect of 4,5-diCQA (20 mg/kg) was comparable to that of diclofenac sodium (10 mg/kg) (Figure 5A). As carrageenan causes acute inflammation, we isolated the spinal cord and performed Western blotting to analyze the expression levels of inflammation-related factors. These findings are consistent with the above paw edema results. Carrageenan injection significantly increased the protein expression of iNOS, COX-2, and TNF-α, and 4,5-diCQA markedly decreased carrageenan-induced protein expression. In addition, the inhibitory effect of 4,5-diCQA (20 mg/kg) on the protein expression of iNOS, COX-2, and TNF-α was comparable to that of diclofenac sodium (10 mg/kg). These results suggest that 4,5-diCQA has a potent anti-inflammatory effect.

## 4. Discussion

The inflammatory response occurs through a series of reactions between immune cells and chemokines. Ultimately, through the normal inflammatory response, the inflammatory response gradually diminished when the antigen is removed [1,2,3]. However, abnormally regulated and sustained inflammatory responses cause secondary illnesses and lead to chronic inflammatory diseases such as asthma and arthritis if not properly treated [1,2,6,7,8]. Although anti-inflammatory agents effectively suppress inflammation, their use is restricted because they cause side effects, such as gastrointestinal disorders [9,10]. This requires the discovery and development of novel anti-inflammatory agents, and the use of natural ingredients is considered as a safer and more effective strategy. In this study, we evaluated the anti-inflammatory effects of 4,5-diCQA in RAW264.7 cells stimulated with LPS and in a rat model of carrageenan-induced paw edema.

Macrophages can mitigate and promote various diseases and conditions such as cancer, atherosclerosis, and inflammation [21,22]. Inflammatory responses in scar tissue induce the activation of macrophages and overexpression of inflammatory mediators (iNOS, COX-2, PGE_2_, and nitrite oxide) and cytokines (TNF-α, IL-6, and IL-1β) [1,3,4,21]. In addition, the expression of these factors is regulated by the NF-κB and MAPK pathways, which are well-known regulatory signaling pathways associated with inflammation [22,23,24,25]. Therefore, reducing the expression of inflammation-related factors through inhibition of signaling pathways is a useful strategy for developing anti-inflammatory agents. Here, 4,5-diCQA markedly suppressed the LPS-induced expression of iNOS, COX-2, PGE_2_, nitrite oxide, TNF-α, and IL-6. In addition, 4,5-diCQA inhibited LPS-induced translocation of the NF-κB p65 subunit to the nucleus and phosphorylation of MAPKs (ERK1/2, JNK, and p38) without affecting the total protein level. These results, along with those of other studies analyzing anti-inflammatory effects, support our hypothesis that 4,5-diCQA has anti-inflammatory properties [20,22,23,26,27].

Carrageenan-induced paw edema, first described by Winter et al., is a widely used model for investigating the physiological pathology of local inflammation and testing the potential anti-inflammatory effects of new compounds [28,29]. Carrageenan injection results in neutrophil infiltration and the release of pro-inflammatory cytokines and prostaglandins, ultimately causing paw edema [20,29,30]. The carrageenan-induced inflammatory response is assessed for up to 6 h because of depletion of the inflammatory cofactor kininogen [28,29,30]. In the early phase (0–3 h after injection), the inflammatory response is mediated by the release of inflammatory mediators such as histamine, 5-HT, and serotonin, whereas the late phase (3–5 h after injection) is mediated by the release of bradykinin, TNF-α, and leukotrienes and is sustained by increased levels of COX-2 and PGE_2_. Commercially available anti-inflammatory drugs target the late phase [28,29,30,31].

In our in vivo study, oral administration of 4,5-diCQA dose-dependently reduced carrageenan-induced paw edema, which was observed in the late phase. Western blot analysis of the spinal cord revealed that carrageenan-induced expression of inflammatory mediators and pro-inflammatory cytokines was decreased in a dose-dependent manner by oral administration of 4,5-diCQA, suggesting that the anti-inflammatory mechanism of 4,5-diCQA is associated with inhibition of inflammatory mediators.

## 5. Conclusions

Pretreatment with 4,5-di-CQA effectively inhibited LPS-induced expression of nitrite oxide, PGE_2_, COX-2, iNOS, TNF-α, and IL-6 via the NF-κB and MAPK signaling pathways. Furthermore, orally administered 4,5-diCQA suppressed carrageenan-induced paw edema in concentration- and time-dependent manners. These results suggest that 4,5-diCQA has anti-inflammatory activity. Therefore, 4,5-diCQA shows potential as an anti-inflammatory agent and functional food ingredient.

## Data Availability

The datasets used and/or analyzed during the current study are available from the corresponding author upon reasonable request.
